# Supplementary material for: Predicting visual function by interpreting a neuronal wiring diagram
Source: Nature. 2024 Oct 2;634(8032):113–23. doi: 10.1038/s41586-024-07953-5 (PMC11446822; doi:10.1038/s41586-024-07953-5)
Supplement: Supplementary file 6 — CRF and ERF predictions for individual TmY4 and TmY9 cells. Analogous to Supplementary Data 3, but for TmY target types. Shown are the top four monosynaptic pathways, the strongest pathway passing through each of the top ten intermediary types (ranking from Extended Data Fig. 7), and the trisynaptic pathway Tm1–TmY–Dm3–TmY (see the section entitled Prediction of spatial normalization). [file 41586_2024_7953_MOESM6_ESM.zip › DataS4/TmY9qΓèÑ/720575940629941820.pdf]

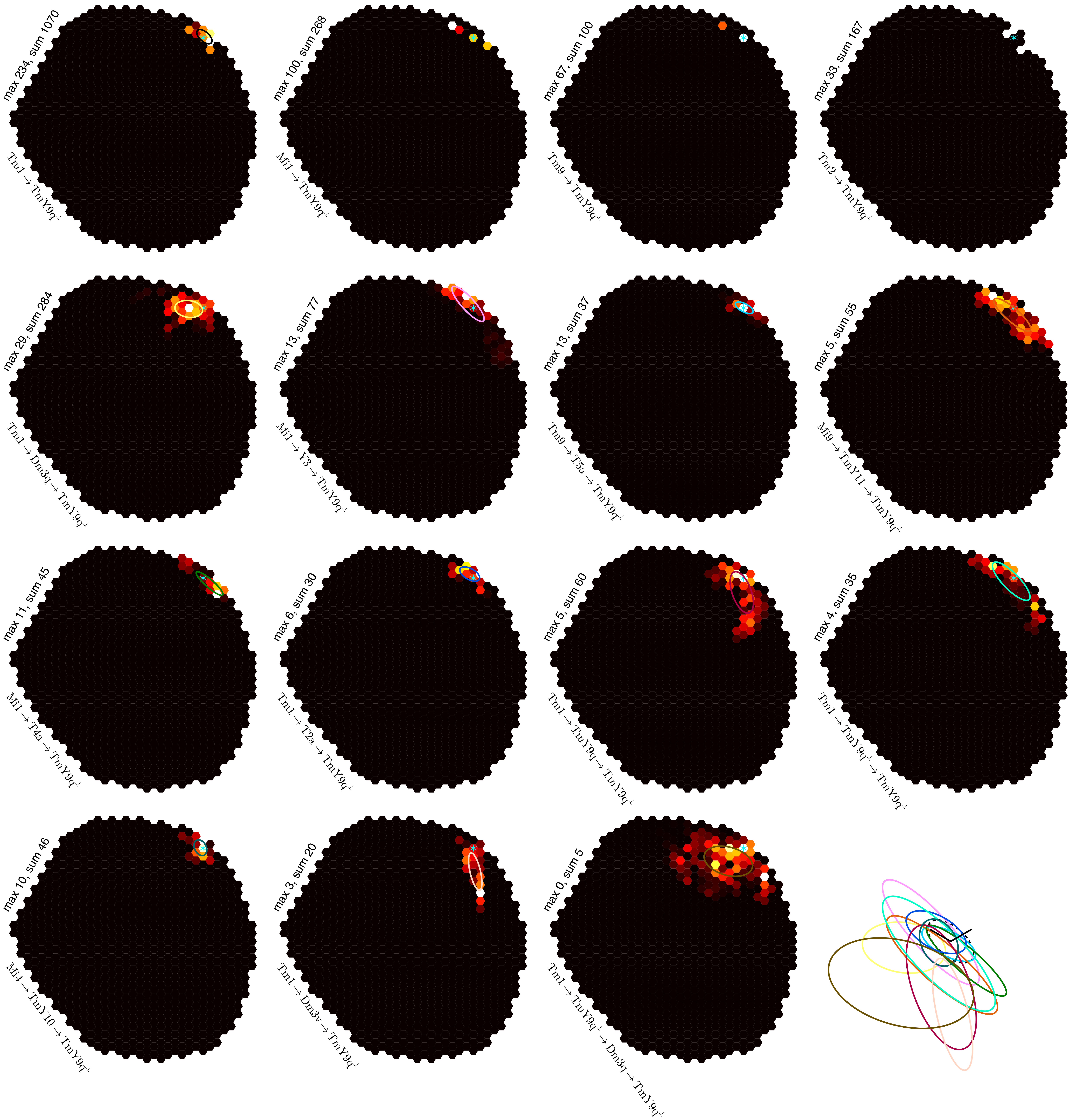
max 234, sum 1070
Tm1 → TmY9q⊥
max 100, sum 268
Mi1 → TmY9q⊥
max 67, sum 100
Tm9 → TmY9q⊥
max 33, sum 167
Tm2 → TmY9q⊥
max 29, sum 284
Tm1 → Dm3q → TmY9q⊥
max 13, sum 77
Mi1 → Y3 → TmY9q⊥
max 13, sum 37
Tm9 → T5a → TmY9q⊥
max 5, sum 55
Mi9 → TmY11 → TmY9q⊥
max 11, sum 45
Mi1 → T4a → TmY9q⊥
max 6, sum 30
Tm1 → T2a → TmY9q⊥
max 5, sum 60
Tm1 → TmY9q → TmY9q⊥
max 4, sum 35
Tm1 → TmY9q⊥ → TmY9q⊥
max 10, sum 46
Mi4 → TmY10 → TmY9q⊥
max 3, sum 20
Tm1 → Dm3v → TmY9q⊥
max 0, sum 5
Tm1 → TmY9q⊥ → Dm3q → TmY9q⊥
